# Pulse Therapy With Corticosteroids and Intravenous Immunoglobulin in the Management of Severe Tocilizumab-Resistant COVID-19: A Report of Three Clinical Cases

**DOI:** 10.7759/cureus.9038

**Published:** 2020-07-07

**Authors:** Mikhail V Sheianov, Yurii D Udalov, Sergei S Ochkin, Andrei N Bashkov, Aleksandr S Samoilov

**Affiliations:** 1 Department of Pulmonology, Burnasyan Federal Medical Biophysical Center of Federal Medical Biological Agency of Russia, Moscow, RUS; 2 Management, Burnasyan Federal Medical Biophysical Center of Federal Medical Biological Agency of Russia, Moscow, RUS; 3 Department of Anesthesiology and Critical Care, Burnasyan Federal Medical Biophysical Center of Federal Medical Biological Agency of Russia, Moscow, RUS; 4 Department of Radiology, Burnasyan Federal Medical Biophysical Center of Federal Medical Biological Agency of Russia, Moscow, RUS

**Keywords:** sars-cov-2, covid-19, pulse therapy, methylprednisolone, immunoglobulin, corticosteroids, cytokine release syndrome

## Abstract

We present the cases of three patients with severe, life-threatening coronavirus disease 2019 (COVID-19) who had failed to achieve substantial improvement on intial treatment. They subsequently received pulse therapy with methylprednisolone [1,000 mg/day intravenously (IV) for three consecutive days] and IV immunoglobulin (20 g/day). This treatment regimen was associated with a prompt resolution of respiratory failure, elimination of clinical manifestations of the cytokine release syndrome (CRS), and reversal of pulmonary CT changes. The treatment was generally safe and well-tolerated. There was no evidence of protracted persistence of the virus in the patients. Further randomized controlled trials are required to better understand the efficacy and safety of high-dose methylprednisolone and IV immunoglobulin, separately or in combination with each other, in the treatment of severe, life-threatening COVID-19.

## Introduction

Clinical presentations of coronavirus disease 2019 (COVID-19) range from asymptomatic cases and mildly symptomatic flu-like forms (81%) to severe (14%) and critical (5%) disease manifesting with pneumonia, hepatic, cardiac, and other organ involvement [1]. Respiratory failure and acute respiratory distress syndrome (ARDS) are common complications [2]. Multiorgan failure and disseminated intravascular coagulation (DIC) can also be observed [2,3]. The aforementioned problems determine the need for intensive care in 17-20% of hospitalized patients [4]. The main cause of death in infected patients worldwide is a combination of both ARDS and DIC leading to a fatal outcome in 11-15% of hospitalized patients [4,5]. The average COVID-19-associated mortality rate has reached 3.7% of reported cases globally [4].

There is growing evidence that the severe course and threatening complications of COVID-19 are caused by excessive and aberrant host immune response induced by the virus in predisposed persons [5,6]. In this context, it seems reasonable to consider treatment involving immunosuppressive drugs [glucocorticoids, intravenous (IV) immunoglobulin, and/or anti-cytokine agents] for patients with severe COVID-19 who exhibit cytokine release syndrome (CRS). The obvious aim of such a treatment is to prevent or reverse the dramatic inflammatory pathway triggered by the virus.

Recently, a number of publications have indicated the positive role that specific interleukin-1 (IL-1) and interleukin-6 (IL-6) inhibitors (anakinra, tocilizumab) can play in the treatment of severe COVID-19 complicated with respiratory failure [7-9]. However, there exists a number of COVID-19 patients in whom it is not possible to achieve a sustained improvement with the use of these drugs [9]. Moreover, tocilizumab and other IL blockers belong to a group of relatively expensive drugs, and this may hamper their use in wide-scale epidemics or in less prosperous healthcare systems. These issues have motivated us to look for alternative options for the treatment of severe, life-threatening cases of COVID-19.

In this report, we describe three cases of severe COVID-19 successfully treated with a combination of methylprednisolone pulse therapy and IV immunoglobulin.

## Case presentation

Case 1

**Figure 1:**
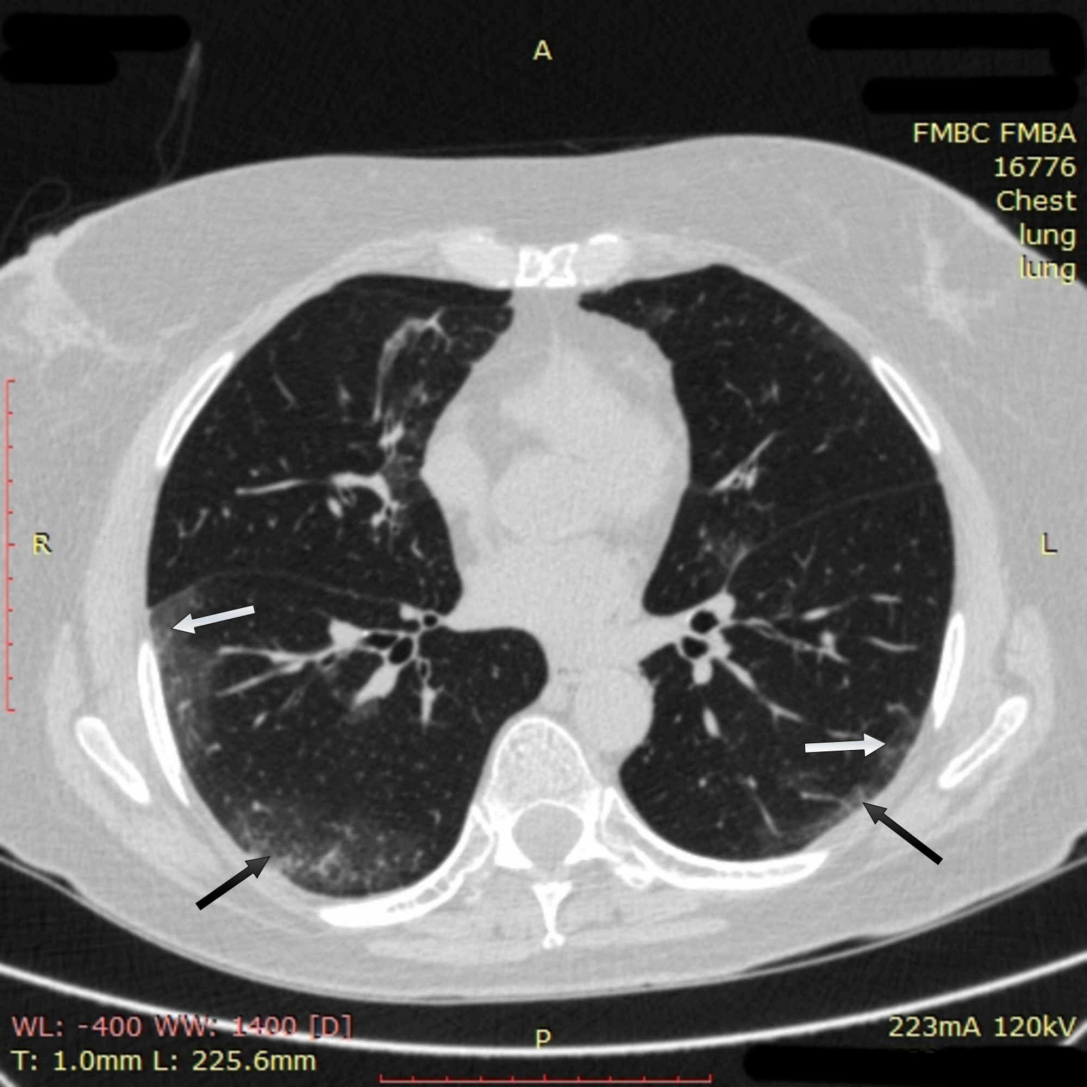
Chest CT imaging of patient 1 on admission CT imaging on admission showed multiple bilateral ground-glass opacities (white arrows) in subpleural areas of lower and middle regions of both lungs; reticular changes in the same zones were also observed (black arrows) CT: computed tomography

A 64-year-old woman with obesity and a history of moderately severe asthma fully controlled on medication presented on April 6, 2020, with six days of fever of up to 38.8 °C, dry exhausting cough, and shortness of breath with little exercise. On examination, she was normothermic (36.2 °C), in moderate respiratory distress, and had oxyhemoglobin desaturation of 94% on room air. CT imaging of the chest on admission revealed multiple bilateral ground-glass opacities, predominantly in subpleural areas of lower and middle regions of both lungs. Reticular changes were also seen in the same zones (Figure 1).

Laboratory tests were mostly normal except for a moderate C-reactive protein (CRP) increase (11.58 mg/L). Procalcitonin level on admission was normal (<0.5 ng/ml) (Table 1).

**Table 1:** Laboratory results of patient 1 WBC: white blood cell count; AST: aspartate aminotransferase (serum glutamic-oxaloacetic transaminase); ALT: alanine aminotransferase (serum glutamic pyruvic transaminase); SARS-CoV-2: severe acute respiratory syndrome coronavirus 2; CRP: C-reactive protein; RNA: ribonucleic acid

Laboratory parameter	Units	Reference range	On admission	Day 11	Day 14	Day 17	Day 22
WBC	x10^9^/L	4.0-9.0	5.7	9.3	10.1	12.2	10.9
Neutrophils (absolute)	x10^9^/L	1.7-7.7	3.8	7.2	8.3	11.3	8.5
Lymphocytes (absolute)	x10^9^/L	0.4-4.4	1.5	1.5	1.1	0.8	2.0
Hemoglobin	g/L	130-170	135	117	115	120	112
Platelets	x10^9^/L	120-380	198	408	426	421	231
ALT	U/L	5-33	15	20	53	45	22
AST	U/L	5-32	23	26	68	61	53
Total bilirubin	mmol/L	5.0-21.0	6.0	-	9.0	12.0	4.0
Creatinine	mmol/L	44-80	61	46	51	49	38
Glucose	mmol/L	3.9-6.0	4.8	5.0	7.1	7.7	5.6
Sodium	mmol/L	136-145	142	142	143	147	143
Potassium	mmol/L	3.5-5.1	4.3	3.7	3.56	3.2	3.9
D-dimer	mg/L	0.00-0.55	-	1.77	-	1.52	-
CRP	mg/L	0.00-5.00	11.58	52.63	54.15	7.91	1.47
Procalcitonin	ng/mL	0.00-0.50	<0.5	0.05	<0.05	0.08	0.03
SARS-CoV-2 RNA	Positive, negative	Negative	Positive	-	-	Positive	Negative

Based on typical symptoms, CT changes in the lungs, and a positive swab test for severe acute respiratory syndrome coronavirus 2 (SARS-CoV-2), the patient was diagnosed with a severe form of COVID-19 complicated with bilateral multilobar pneumonia and acute respiratory failure. The patient was started on clarithromycin 1,000 mg/day orally (PO) and hydroxychloroquine 800 mg PO on the first day followed by a dose reduction to 400 mg daily from the second day, ceftriaxone 2,000 mg/day IV, and tilorone 125 mg PO daily for antiviral treatment. Oxygen supplementation via a facial mask (5 L/min) was initiated leading to an increase in oxygen saturation (SpO_2_) level in the patient to 96% with improvement in respiratory discomfort.

On April 9, 2020 (day four of hospitalization), the patient’s condition began to deteriorate with increasing dyspnea and progressive fall in SpO_2_ from 93% on day four to 90% by day 11 despite noninvasive low-flow oxygenation of 5 L/min via facial mask. The patient was switched to an alternative treatment including antiviral agents lopinavir 800 mg PO daily, ritonavir 200 mg PO daily, and umifenovir 400 mg PO daily. Antimicrobial therapy was replaced with meropenem 3,000 mg IV daily.

**Figure 2:**
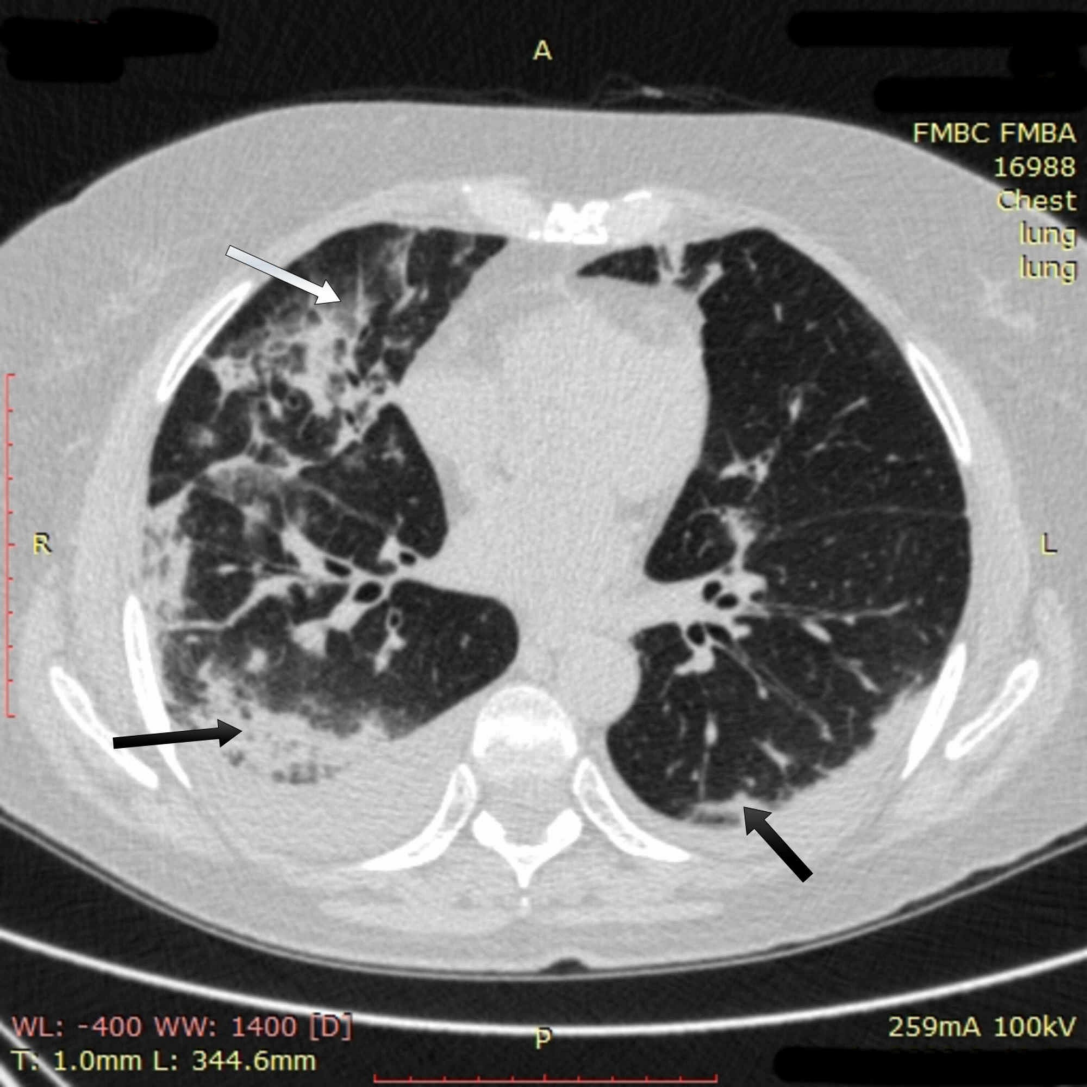
Chest CT imaging of patient 1 on day 11 Chest CT on day 11 showed multiple bilateral ground-glass opacities in subpleural and central regions of the middle and upper right lobes with a noticeable reticular pattern (white arrow); subpleural areas of consolidation in both lungs (black arrows) were also seen CT: computed tomography

Despite the measures taken, no positive change in the patient was observed by day 11. Chest CT on day 11 showed multiple bilateral ground-glass opacities with new obfuscation zones in subpleural and central regions of the middle and upper right lobes with a noticeable reticular pattern. The density of previously seen opacities increased, and subpleural areas of consolidation appeared in the left lung (Figure 2). Overall involvement of pulmonary tissue reached 75% in the right lung and 30-50% in the left one.

Laboratory data showed a high level of CRP (52.63 mg/L) and an increased concentration of D-dimer (1.77 mg/L). Body temperature and procalcitonin level were normal. Considering a high risk of progression to severe respiratory failure and intubation, a moderate dose of methylprednisolone (125 mg daily IV) was prescribed to the patient and given on days 11-14. Antimicrobial therapy with meropenem was continued.

Despite this, after temporary stabilization, the patient’s condition continued to worsen. On day 14, SpO_2_ decreased to 86% on low-flow oxygenation of 5 L/min via facial mask in a prone position. The patient was fully conscious but suffered from pronounced dyspnea and fatigue. Chest X-ray on day 14 showed diffuse round multiple opacities without definite borders all over the lungs with a tendency to merge. Opacification involved more than 75% of the right lung and at least 50% of the left lung tissue. Laboratory data showed the same changes as on day 11.

Because of her progressive respiratory failure, the patient was transferred to the medical intensive care unit (ICU), where she was started on high-flow oxygen therapy of 45 L/min FiO2 90% via nasal cannula. This raised SpO_2_ to 90-93% with blood oxygen saturation dropping back to 80% on room air. The patient was given a humanized anti-IL-6 receptor antibody, tocilizumab, (400 mg IV) once on day 14. On days 14 and 15, the condition of the patient remained extremely severe, without any improvement. Hypoxemia with SpO_2 _of 88-90% persisted despite high-flow oxygenation of up to 50 L/min FiO_2_ 100% and prone-positioning of the patient.

Considering the severe, life-threatening course of COVID-19 in a high-risk person, the lack of sufficient clinical response to tocilizumab, and an emerging perspective of intubation with subsequent fatality risk of more than 90% [2], the multidisciplinary team decided to prescribe a pulse therapy with methylprednisolone (1,000 mg/day IV for three consecutive days) to the patient as a potentially life-saving compassion treatment. Pulse therapy with methylprednisolone was accompanied by IV immunoglobulin 20 g/day for three consecutive days to prevent steroid-induced immunodeficiency and for its own immunomodulatory effect. This treatment was conducted on hospital days 15-17.

**Figure 3:**
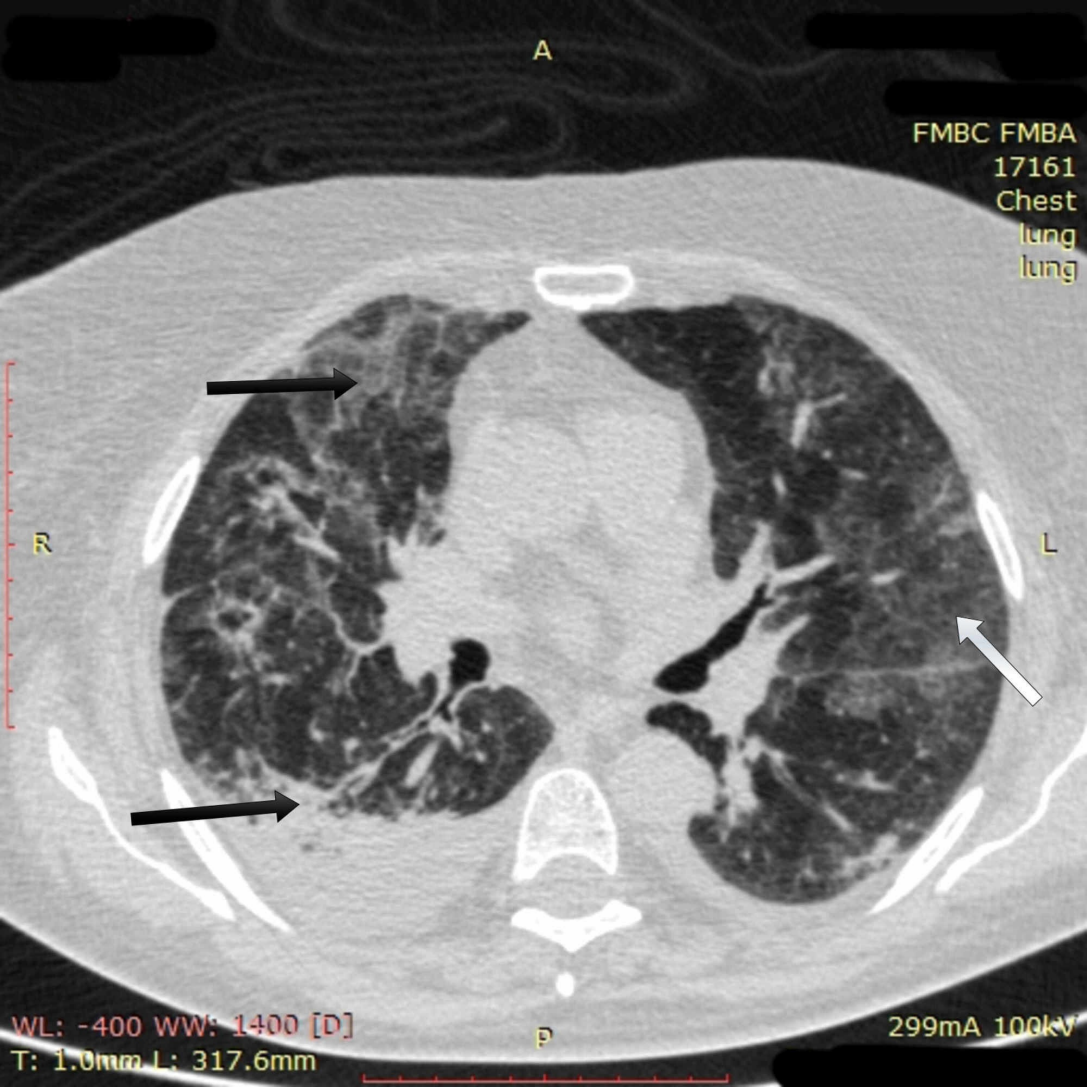
Chest CT imaging of patient 1 on day 17 Chest CT on day 17 showed multiple confluent ground-glass opacities had increased in size (white arrow); bilateral pulmonary infiltrates had lost their density to the degree of ground-glass opacity, and their area had decreased (black arrows) CT: computed tomography

On the evening of day 15, SpO_2_ stabilized in the patient at 95-96% on decreased high-flow oxygen of 10 L/min FiO_2_ 50% with a respiratory rate of 14-15 breaths per minute. On day 16, SpO_2_ reached 99% on high-flow oxygenation of 10 L/min. On day 17, high-flow oxygenation was discontinued, and the patient sustained SpO_2_ at 95-97% on low-flow oxygenation of 5 L/min via facial mask. Chest CT imaging on day 17 showed multidirectional changes. Multiple confluent ground-glass opacities increased in size. However, initial positive change was also found: bilateral pulmonary infiltrates had lost their density to the degree of ground-glass opacity and their area had decreased (Figure 3).

**Figure 4:**
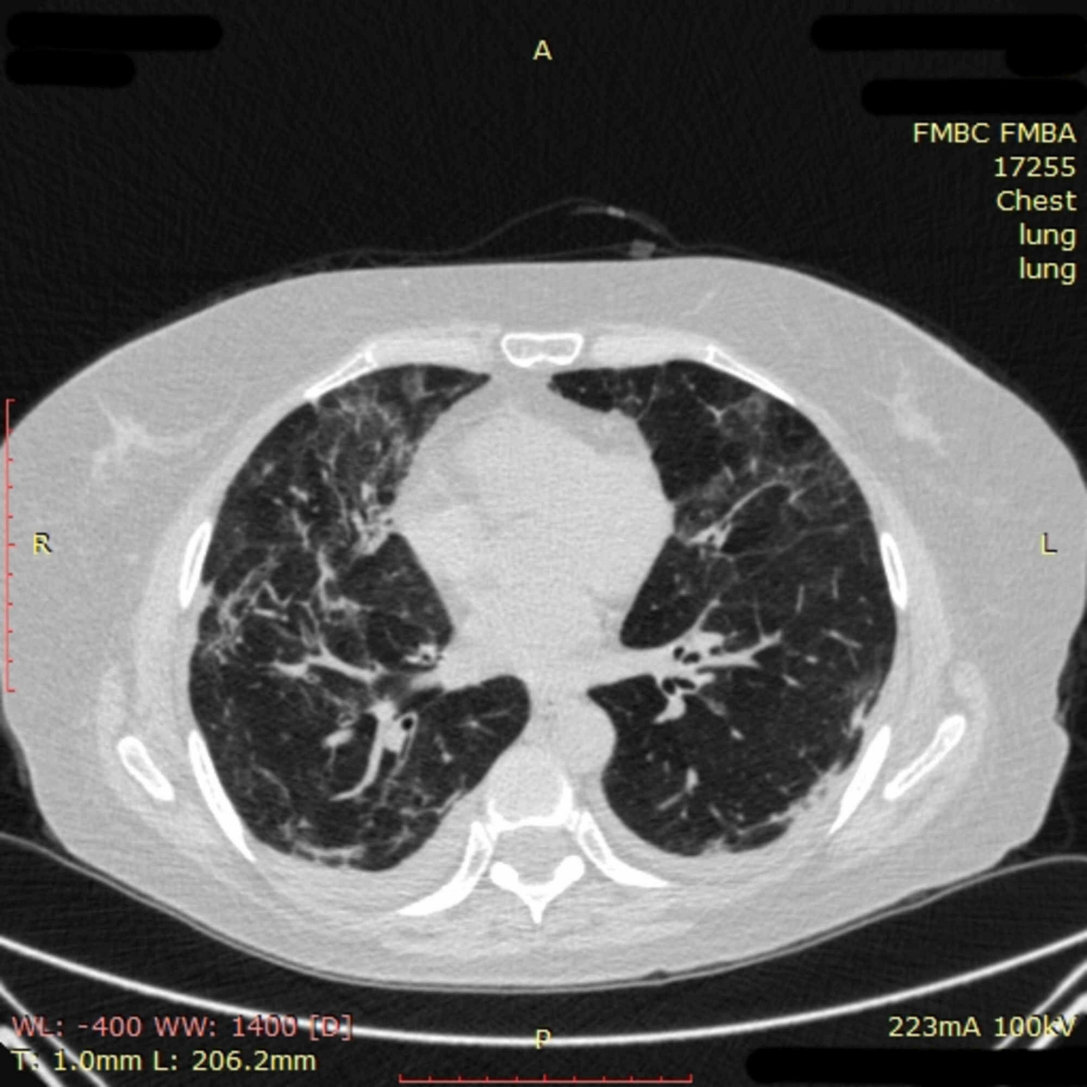
Chest CT imaging of patient 1 on day 22 Chest CT on day 22 showed that opacification in the left lung had mostly resolved, and the area and density of obfuscation in the right lung had clearly decreased CT: computed tomography

Further positive changes were seen on CT images on day 22: opacification in the left lung mostly resolved, and the area and density of obfuscation in the right lung had clearly decreased (Figure 4).

During the following days, the clinical condition of the patient continued to improve. Respiratory failure resolved, and the patient was able to sustain normal (97-98%) blood oxygen saturation on room air by day 25. Respiratory rate, blood pressure, and heart rate also returned to normal values. Respiratory symptoms decreased, leaving a small degree of dyspnea and fatigue. The first SARS-CoV-2-negative nasopharyngeal swab was obtained on day 20 and confirmed two days later. The patient was declared to be cured and discharged from the hospital on May 5, 2020 (hospital day 30).

Case 2

**Figure 5:**
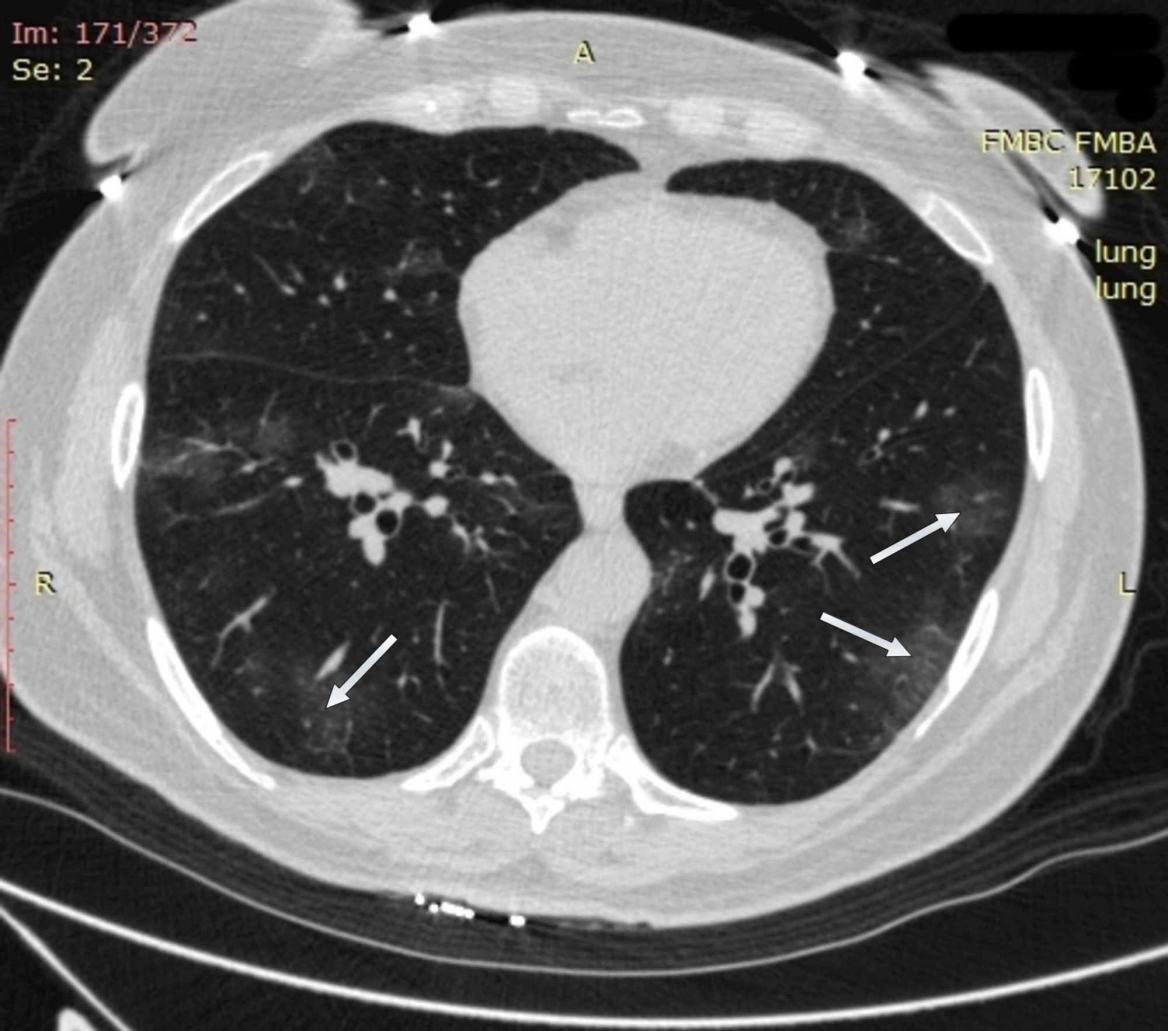
Chest CT imaging of patient 2 on admission Chest CT imaging on admission revealed multiple ground-glass opacities of up to 3 cm in size (white arrows) in the majority of segments of both lungs, predominantly in the lower and middle regions, affecting less than 50% of the lung parenchyma CT: computed tomography

On April 20, 2020, a 60-year-old woman with obesity and a history of chronic pancreatitis presented with five days of fever, dry cough, and weakness. On examination, she was febrile with 38.8 °C, not in respiratory distress, and her peripheral SpO_2_ was 95% on room air. A CT scan of the lungs on admission revealed multiple bilateral ground-glass opacities (Figure 5).

COVID-19 was confirmed by a positive polymerase chain reaction (PCR) test obtained via a nasopharyngeal swab, and she was started on hydroxychloroquine, clarithromycin, ceftriaxone, and tilorone.

On day three, the patient's condition deteriorated with the appearance of dyspnea at rest and aggravation of fever and chills. Oxyhemoglobin saturation decreased to 87-91% on room air, requiring noninvasive low-flow oxygenation via facial mask. A CT scan on day three revealed large subpleural ground-glass opacities affecting up to 50% of the lung parenchyma. Laboratory tests demonstrated a high level of CRP and ferritin (Table 2).

**Table 2:** Laboratory results of patient 2 WBC: white blood cell count; AST: aspartate aminotransferase (serum glutamic-oxaloacetic transaminase); ALT: alanine aminotransferase (serum glutamic pyruvic transaminase); SARS-CoV-2: severe acute respiratory syndrome coronavirus 2; CRP: C-reactive protein; RNA: ribonucleic acid

Laboratory parameter	Units	Reference range	On admission	Day 3	Day 8	Day 10	Day 21
WBC	x10^9^/L	4.0-9.0	3.3	4.3	8.6	4.8	4.0
Neutrophils (absolute)	x10^9^/L	1.7-7.7	1.7	3.1	7.7	3.5	2.3
Lymphocytes (absolute)	x10^9^/L	0.4-4.4	1.1	0.9	0.8	1.2	1.4
Hemoglobin	g/L	130-170	128	121	111	110	116
Platelets	x10^9^/L	120-380	176	163	279	305	271
ALT	U/L	5-33	37	101	93	100	126
AST	U/L	5-32	38	87	55	80	51
Total bilirubin	mmol/L	5.0-21.0	4.0	4.0	7.0	7.0	8.9
Creatinine	mmol/L	44-80	48	50	43	45	48
Glucose	mmol/L	3.9-6.0	6.0	7.1	7.5	9.0	5.9
Sodium	mmol/L	136-145	141	-	140	136	142
Potassium	mmol/L	3.5-5.1	4.2	-	3.4	3.2	3.8
D-dimer	mg/L	0.00-0.55	1.36	0.75	0.92	1.54	0.42
CRP	mg/L	0.00-5.00	7.52	34.77	17.34	5.80	0.69
Procalcitonin	ng/mL	0.00-0.50	0.05	0.04	0.02	<0.5	-
Ferritin	ng/mL	28-365	-	868	797	-	-
SARS-CoV-2 RNA	Positive, negative	Negative	Positive	Positive	Positive	Positive	Negative

Considering the progression of the disease and significantly elevated markers of inflammation, the patient was diagnosed with CRS. A moderate dose of corticosteroids (methylprednisolone 125 mg/day IV) and enoxaparin 40 mg subcutaneously twice daily were added to her therapy. During the next four days, the condition of the patient improved; her temperature decreased to 37.2 °C and she was no longer in respiratory distress.

**Figure 6:**
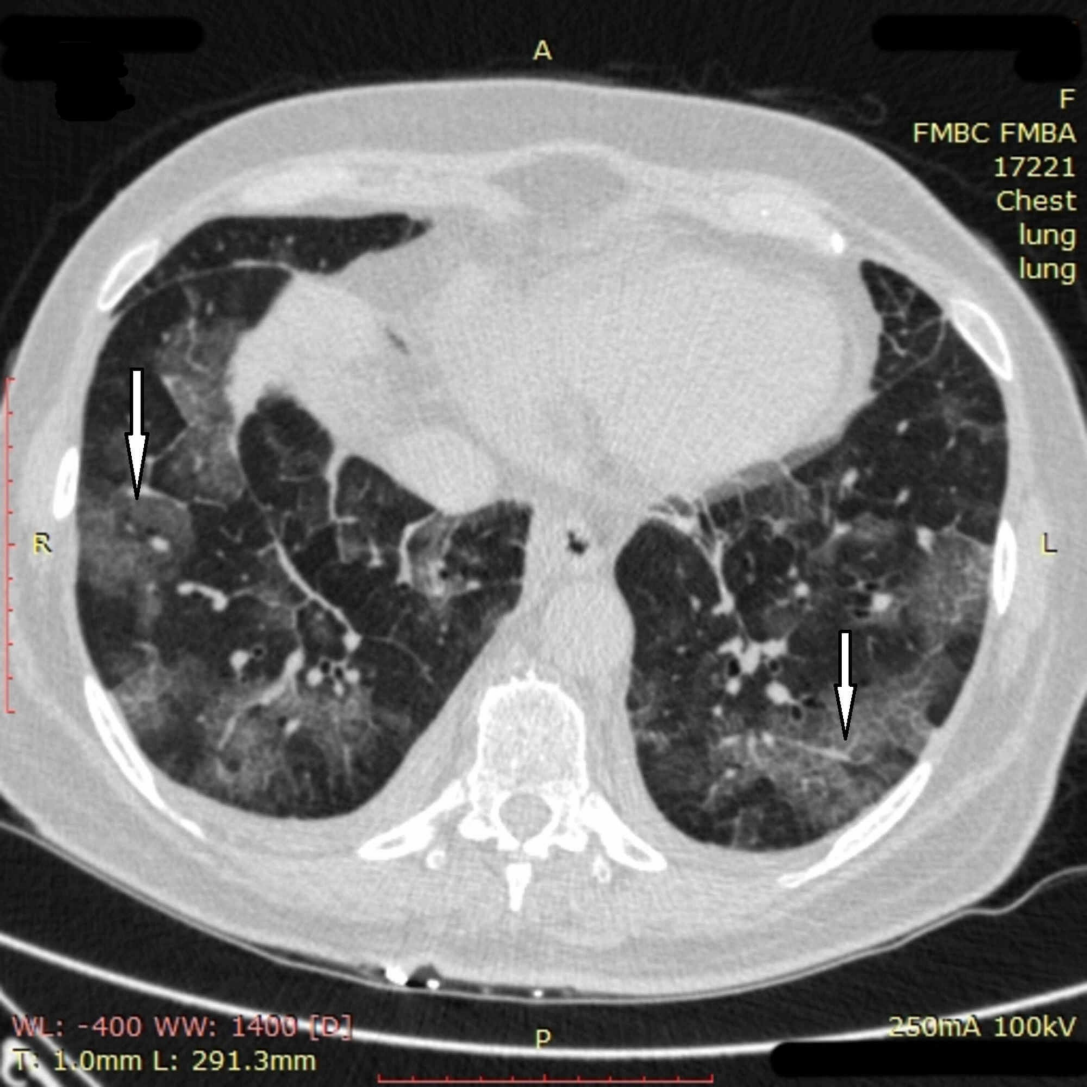
Chest CT imaging of patient 2 on day eight CT scan on day eight revealed an increase of pre-existing opacities in size and density. New ground-glass opacities appeared in upper and lower zones of the lungs merging into large subpleural opacification areas (white arrows). In the left lung, small foci of consolidation were seen. Interlobular septal thickening could be noted in various parts of the lungs. Overall, pathological changes involved at least 75% of pulmonary tissue CT: computed tomography

However, on day seven, the patient developed fever up to 39.3 °C and a fall in SpO_2_ to 88% on low-flow oxygenation of 5 L/min via facial mask. The patient was transferred to the medical ICU where she was started on high-flow oxygenation of 40 L/min FiO2 50% via nasal cannula. The patient was also given tocilizumab (400 mg IV). However, for the next three days, she continued to experience persistent fever, pronounced dyspnea, and fatigue. It was not possible to raise her SpO_2_ above 94%, and high-flow oxygen of up to 50 L/min FiO_2 _50% was required to reach this. On day eight, a CT scan revealed a further increase of subpleural ground-glass opacities (Figure 6).

**Figure 7:**
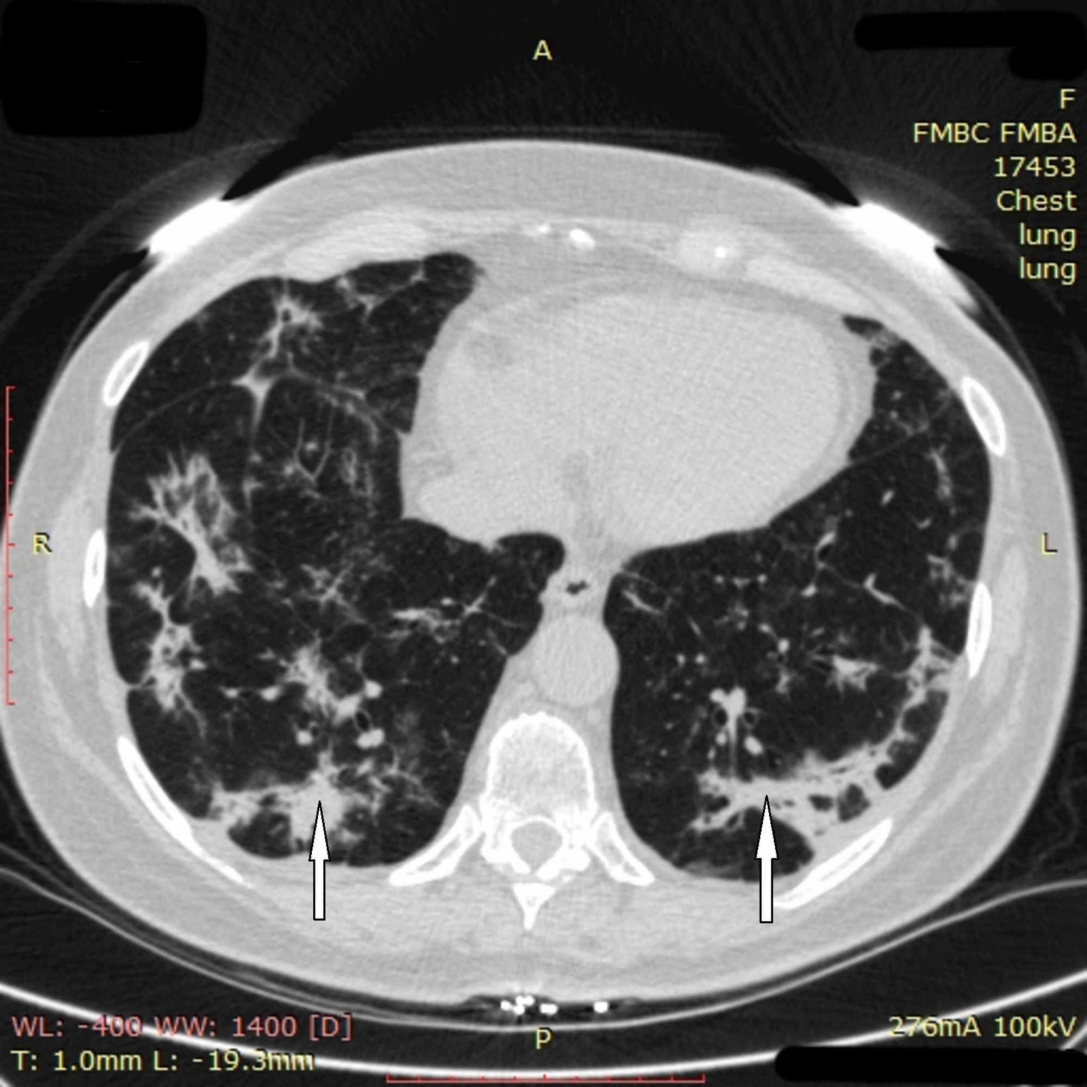
Chest CT imaging of patient 2 on day 13 Chest CT on day 13 showed partial or complete resolution of the opacities in both lungs with the amount of involved pulmonary tissue decreased to less than 50%. A portion of opacities in all segments of the lungs had turned into consolidation (white arrows) CT: computed tomography

Laboratory data showed a high ferritin level and a moderate elevation of CRP, D-dimer, and aminotransferases. Because of the severe, life-threatening course of COVID-19 and insufficient effectiveness of previous treatment, the patient was given a pulse therapy with methylprednisolone (1,000 mg/day IV for three consecutive days) accompanied by IV immunoglobulin (20 g/day). This treatment was conducted on days 10-12. After that, the patient's condition improved significantly. Her body temperature and SpO_2_ normalized; high-flow oxygenation was discontinued, and the patient only had moderate residual weakness and dyspnea. On day 13, she returned to the regular ward and was discharged in good clinical condition on day 23 with two negative swab tests for SARS-CoV-2. Chest CT at discharge showed partial or complete resolution of ground-glass opacities in both lungs. A portion of opacities in all segments had turned into consolidation with no signs of pulmonary infection (Figure 7).

Case 3

**Figure 8:**
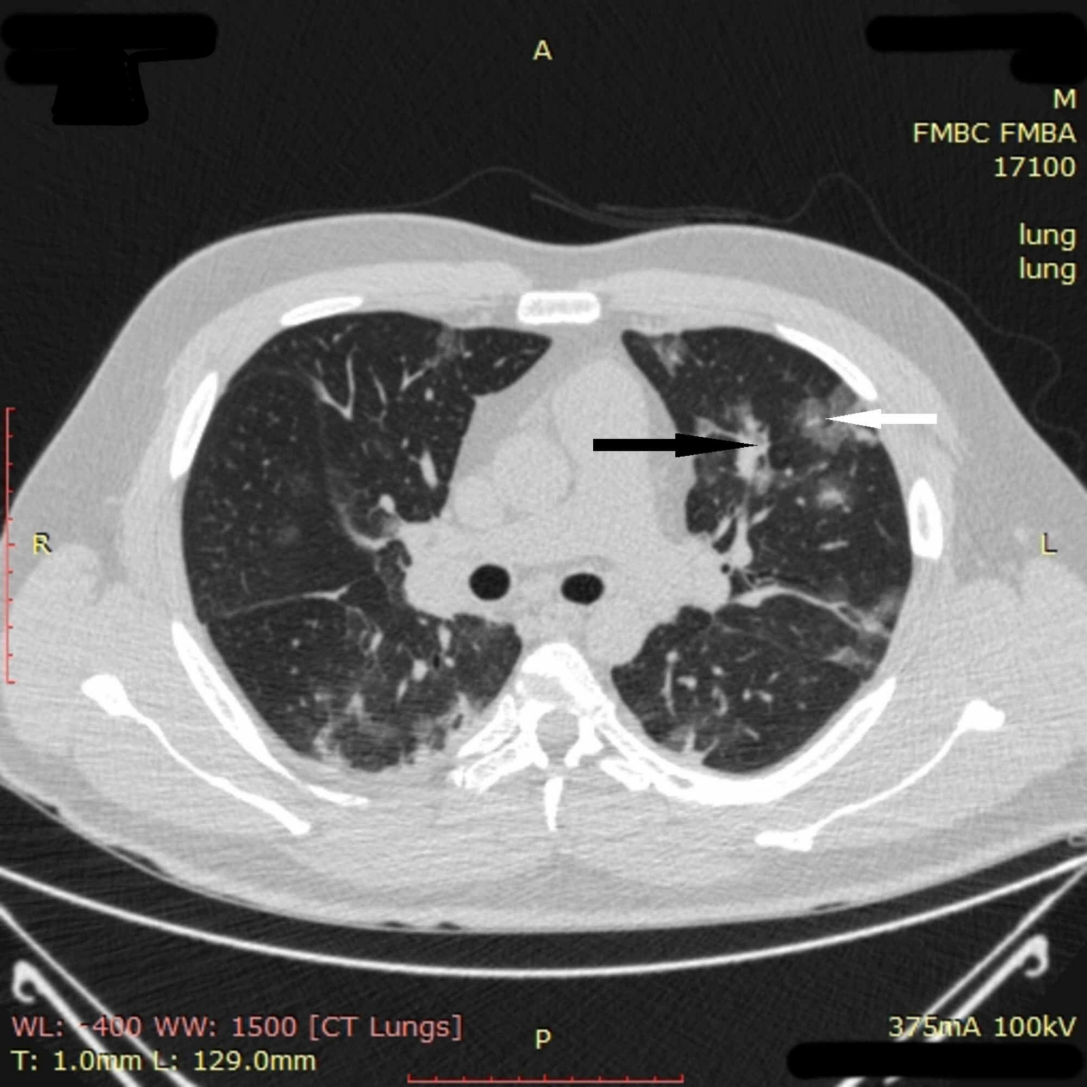
Chest CT imaging of patient 3 on admission Chest CT on admission revealed multiple ground-glass opacities (white arrow) and areas of pulmonary consolidation (black arrow) in all segments of the lungs CT: computed tomography

On April 21, 2020, a 33-year-old man with no medical history presented with seven days of fever, weakness, dyspnea on exertion, and loss of appetite. On examination, he was febrile with 38 °C, in moderate respiratory distress, and had mild oxyhemoglobin desaturation (SpO_2 _of 94% on room air). Chest CT on admission revealed multiple ground-glass opacities and areas of pulmonary consolidation in all segments of the lungs (Figure 8).

Blood tests demonstrated an extra-high level of ferritin and noticeably increased CRP and aminotransferases (Table 3).

**Table 3:** Laboratory results of patient 3 WBC: white blood cell count; AST: aspartate aminotransferase (serum glutamic-oxaloacetic transaminase); ALT: alanine aminotransferase (serum glutamic pyruvic transaminase); SARS-CoV-2: severe acute respiratory syndrome coronavirus 2; CRP: C-reactive protein; RNA: ribonucleic acid

Laboratory Parameter	Units	Reference range	On admission	Day 4	Day 9	Day 17	Day 23
WBC	x10^9^/L	4.0-9.0	9.7	9.9	34.7	5.9	5.1
Neutrophils (absolute)	x10^9^/L	1.7-7.7	7.80	8.40	33.20	3.90	2.80
Lymphocytes (absolute)	x10^9^/L	0.4-4.4	1.50	1.30	1.20	1.50	2.0
Hemoglobin	g/L	130-170	154	145	114	117	138
Platelets	x10^9^/L	120-380	206	230	331	223	250
ALT	U/L	5-33	124	160	61	65	54
AST	U/L	5-32	161	156	57	31	30
Total bilirubin	mmol/L	5.0-21.0	4.0	8.0	11.0	7.0	13.5
Creatinine	mmol/L	44-80	85	86	122	71	87
Glucose	mmol/L	3.9-6.0	5.8	9.4	8.5	5.9	5.0
Sodium	mmol/L	136-145	142	141	136	140	141
Potassium	mmol/L	3.5-5.1	4.2	4.0	4.6	4.5	4.3
D-dimer	mg/L	0.00-0.55	1.41	0.49	1.34	1.29	-
CRP	mg/L	0.00-5.00	44.64	2.85	119.67	8.25	2.08
Procalcitonin	ng/mL	0.00-0.50	0.08	<0.50	>2	<0.50	<0.50
Ferritin	ng/mL	28-365	2540	1140	-	644	-
SARS-CoV-2 RNA	Positive, negative	Negative	Positive	Positive	Positive	Positive	Negative

COVID-19 was confirmed by a positive PCR test obtained via a nasopharyngeal swab. Because of a pronounced pulmonary involvement on CT and clinical data suggestive of CRS, the patient was started on methylprednisolone 125 mg IV daily, enoxaparin, lopinavir, ritonavir, umifenovir, and ceftriaxone. Despite treatment, during the first two hospital days, he experienced severe symptoms including transient elevations of body temperature to 38 °C with chills, cough, shortness of breath, dizziness, and insomnia. Blood oxygen saturation decreased to 85-90% on room air, requiring noninvasive low-flow oxygenation with growth in SpO_2_ to 90-92% on 5 L/min of oxygen via facial mask. The patient was fully conscious, complied with recommendations of the personnel, and was put to a prone-position for two hours every two hours.

On day three, he underwent a deterioration with severe dyspnea at rest, aggravation of fever (38.1 °C), and fall in SpO_2_ to 87% on low-flow oxygenation of 5 L/min via facial mask. The patient was transferred to the medical ICU where he was started on high-flow oxygenation of 40 L/min FiO_2_ 50% via nasal cannula and given 400 mg tocilizumab IV. By the end of day three, the body temperature decreased in the patient to 37.1 °C. On high-flow oxygenation via nasal cannula, blood oxygen saturation fluctuated around 92% with rapid desaturation to 87% on room air; the respiratory rate was 22-24 breaths per minute. The patient was hemodynamically stable with a blood pressure of 130/70 mmHg and a heart rate of 94 beats per minute.

**Figure 9:**
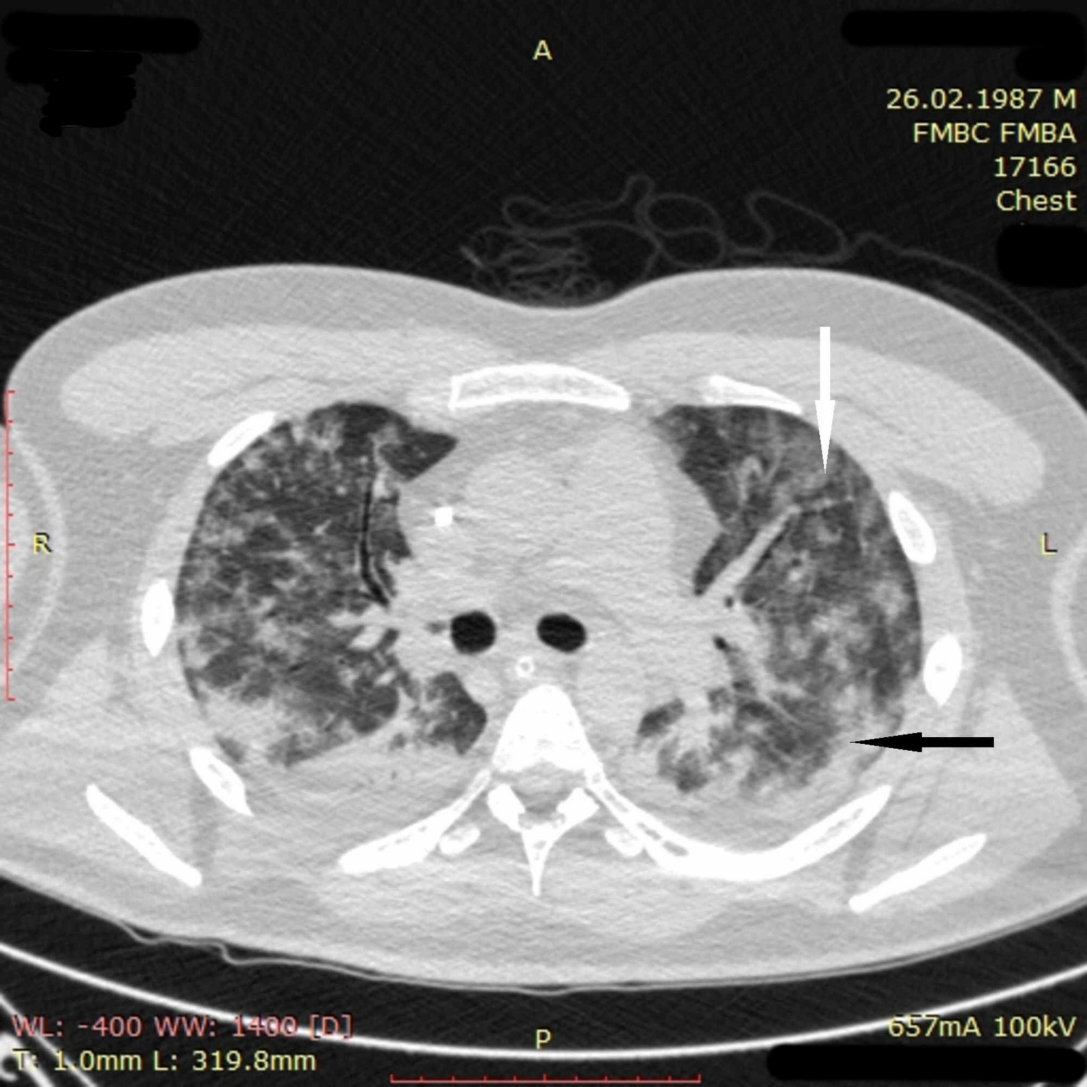
Chest CT imaging of patient 3 on day four CT scan on day four demonstrated the appearance of multiple merging ground-glass opacities (white arrow) and areas of consolidation (black arrow) in all segments of the lungs. Partial atelectasis of both lower lobes was also noted. In general, pathological changes involved more than 75% of pulmonary tissue. CT picture was suggestive of ARDS CT: computed tomography; ARDS: acute respiratory distress syndrome

On day four, a threatening progression of respiratory insufficiency occurred. There was a registered a fall in SpO_2_ to 80% on high-flow oxygenation via nasal cannula; the patient became agitated, his respiratory rate increased to 42-44 per minute, blood pressure reached 175/94 mmHg, and heart rate increased to 135 per minute. Arterial blood gas analysis revealed a pH of 7.33, paO_2 _of 59 mmHg, and paCO_2_ of 35.7 mmHg. The patient was intubated and treated with low tidal volume ventilation. This led to the stabilizing of his clinical condition. Blood oxygen saturation increased to 98%, blood pressure decreased to 120/70 mmHg, and heart rate was 85 per minute. CT scan on day four demonstrated multiple ground-glass opacities and areas of consolidation in all segments of the lungs (Figure 9). CT picture was suggestive of ARDS.

Considering the risk of the unfavorable future course of the disease, the patient was prescribed a pulse therapy with methylprednisolone. On hospital days four to six, he received methylprednisolone 1,000 mg/day IV, and his condition noticeably improved. On day six, sedation was discontinued, and the patient demonstrated SpO_2_ of 98% on spontaneous ventilation. He was fully conscious and responsive to non-verbal contact.

**Figure 10:**
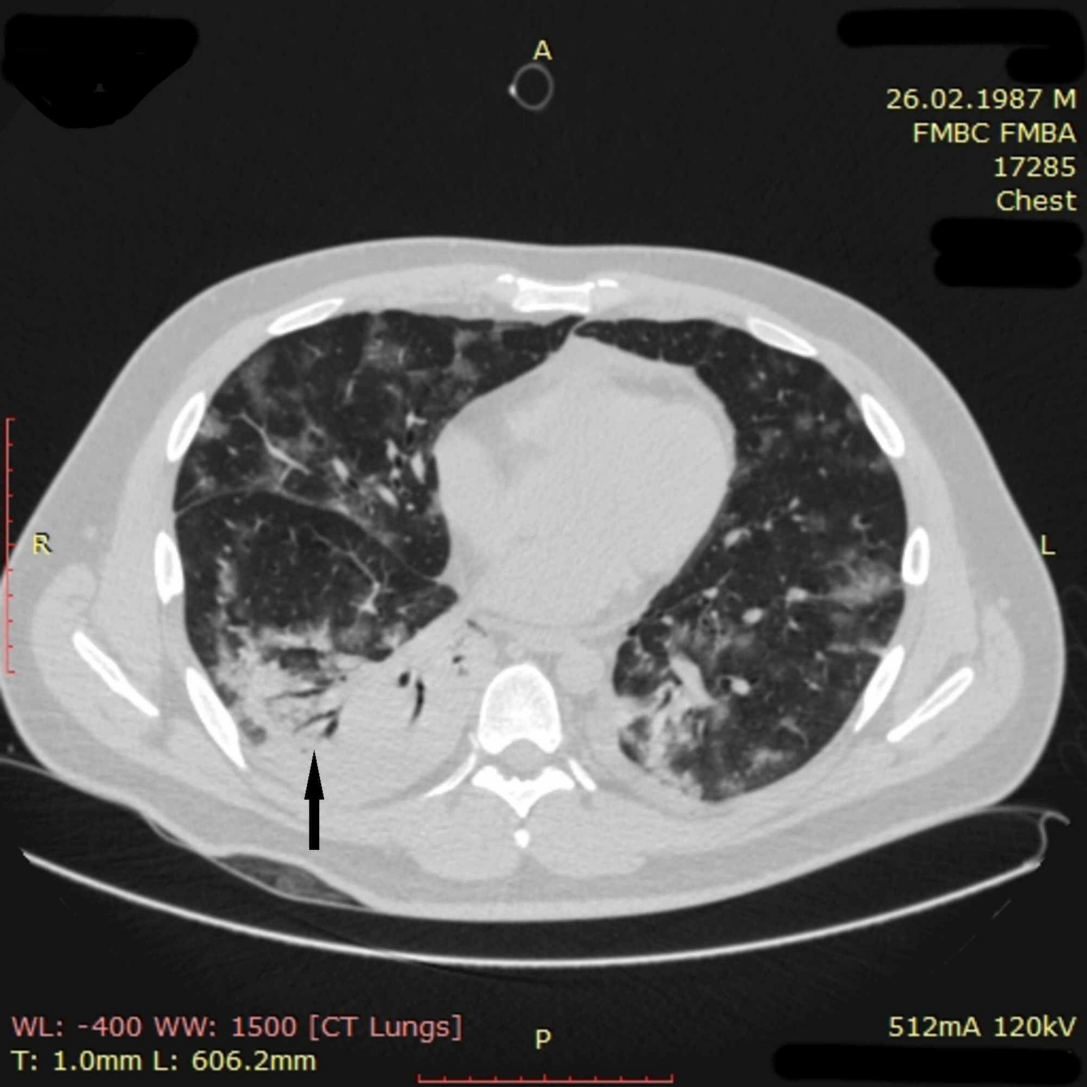
Chest CT imaging of patient 3 on day nine Chest CT performed on day nine showed a distinct positive change in CT picture compared to that of day four. The ground-glass opacities noticeably decreased in amount and density all over the lungs. Pneumatized parenchyma could be seen again in all pulmonary segments. The total volume of affected lung tissue was down to approximately 50%. However, a new zone of pulmonary tissue consolidation with a symptom of air bronchogram (black arrow) was found in the lower lobe of the right lung, suggestive of pneumonia CT: computed tomography

On day eight, the patient experienced transient deterioration with an elevation of body temperature to 40.1 °C, SpO_2 _fall to 94%, and septic shock requiring a norepinephrine drip. Chest CT on day nine showed a distinct positive change in CT picture compared to that of day four. The ground-glass opacities decreased in amount and density all over the lungs. However, new pulmonary tissue consolidation was found in the lower lobe of the right lung, suggestive of pneumonia (Figure 10).

Blood analysis on day nine revealed a pronounced neutrophil leukocytosis, mild normochromic anemia, further elevation of CRP, and a high level of procalcitonin. The patient was diagnosed with nosocomial pneumonia. After a seven-day course of meropenem and an infusion of IV immunoglobulin (20 g), his condition finally improved; he was weaned from the ventilator on day 14 and returned to the regular ward on day 16 with SpO_2 _of 99% on low-flow oxygen.

**Figure 11:**
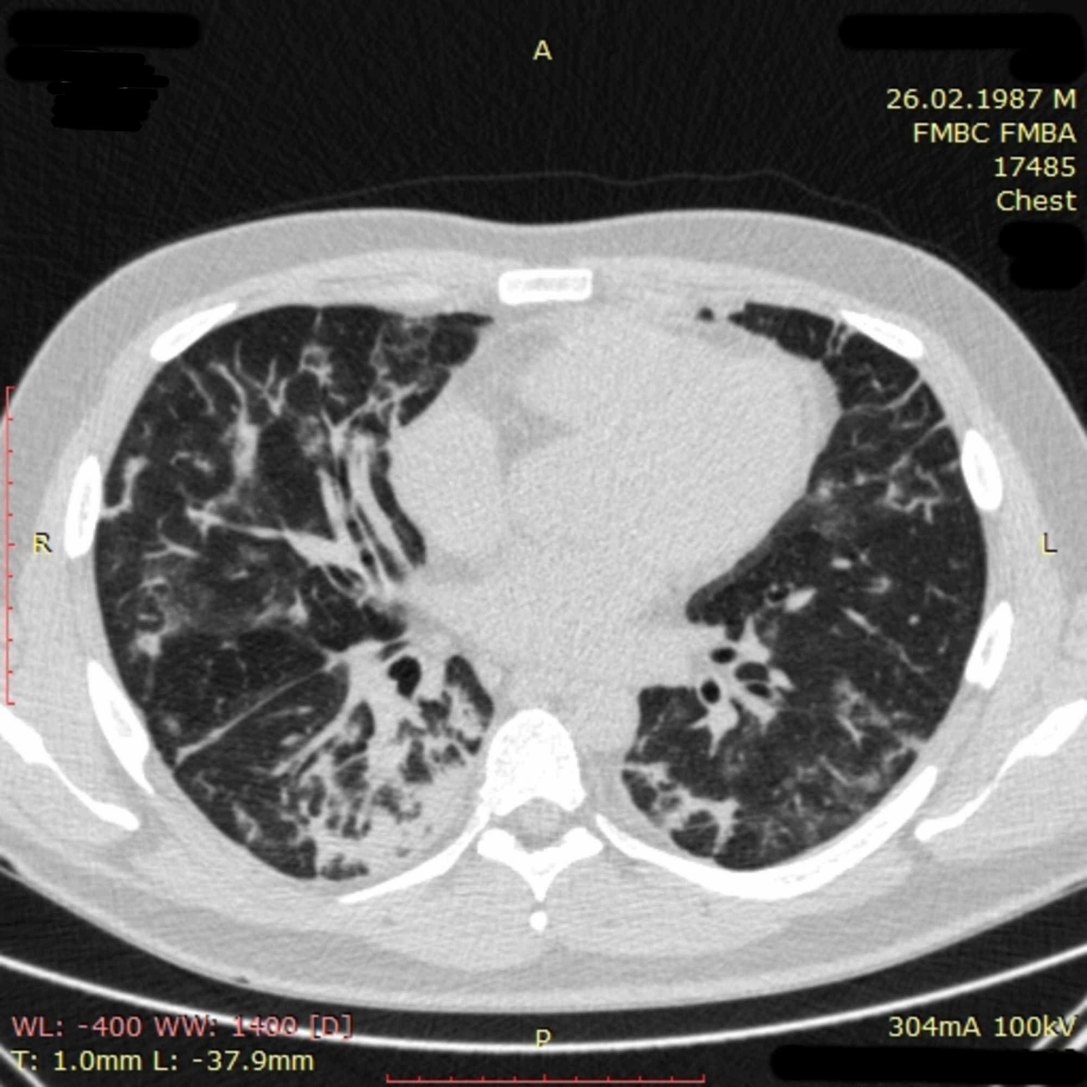
Chest CT imaging of patient 3 on day 23 Chest CT on day 23 revealed partial resolution of pulmonary tissue consolidation in the right lower lobe and a further decrease of size and density of ground-glass opacities all over the lungs CT: computed tomography

The patient was discharged home on day 23 with no symptoms, normal oxyhemoglobin saturation, and a negative nasopharyngeal swab test for SARS-CoV-2. Chest CT on discharge revealed partial resolution of pulmonary consolidation in the right lower lobe and a further decrease in size and density of ground-glass opacities all over the lungs (Figure 11).

## Discussion

In this report, we described a cohort of three patients with severe, life-threatening COVID-19, who had failed to improve after consecutive administration of moderate doses of corticosteroids and specific IL-6 inhibitor tocilizumab, but demonstrated a favorable response to pulse therapy with high doses of methylprednisolone and IV human immunoglobulin. 

In two of the patients, deterioration was associated with clinical features suggestive of hyperinflammation possibly due to CRS [high fever, (multi)organ failure, marked elevation of CRP, ferritin, D-dimer, and aminotransferases]. In one patient, vast pulmonary involvement and respiratory failure were accompanied by a relatively modest increase in the level of CRP and D-dimer. Considering the evidence relating to the immunopathological nature of COVID-19-associated severe pneumonia, a specific IL-6 inhibitor, tocilizumab, was selected to counter the rampant exacerbation in these patients. Unfortunately, tocilizumab did not produce a prompt clinical relief in this cohort.

Patients with severe COVID-19 usually have increased levels of multiple cytokines [10,11]. It can be assumed that the blockade of only one of them, namely IL-6, may not have enough power to suppress the development of such a threatening COVID-19 complication as CRS. To be fully effective in the treatment of CRS in COVID-19 patients, immunomodulating therapies are still awaited to suppress multiple proinflammatory cytokine pathways. And in such situations, corticosteroids along with IV immunoglobulin are usually used first [12]. Facing continued worsening of the condition of the described patients, the multidisciplinary group in charge of treatment decided to use pulse therapy with high doses of corticosteroids and IV immunoglobulin as *ultima ratio* before the obvious indications to intubation occur or immediately after intubation given the unpredictable outcome on mechanical ventilation.

Pulse therapy with methylprednisolone and/or IV immunoglobulin is frequently used in situations where it is necessary to obtain a prompt immunosuppressive effect [13-16]. To our knowledge, pulse therapy with corticosteroids had not been previously used for the treatment of severe lung impairment in COVID-19. And we have managed to find only one report about the successful use of IV immunoglobulin in patients with severe COVID-19 [17]. But we consider such therapy justified as both steroids and IV immunoglobulin are positioned as the first-line cure for CRS [12].

The result seemed overwhelmingly positive to us. During the first day of pulse therapy, the patients' blood oxygen saturation and hemodynamics stabilized, and body temperature decreased if elevated. Till the end of their three-day course, there was no need for high-flow oxygenation any more in two of the patients. The third patient, who was intubated prior to pulse therapy, was switched to spontaneous ventilation and was capable of sustaining a 98% blood oxygen saturation on this regimen. In all the patients, clinical improvement was accompanied by a rapid positive dynamic of the CT picture in the lungs. The patients were soon discharged in good clinical condition with negative swab tests for SARS-CoV-2. Of note, patients with a similar catastrophic course of the disease often die or need prolonged mechanical ventilation with unpredictable outcomes [2].

Pulse therapy with methylprednisolone and IV immunoglobulin was generally safe and well-tolerated. We did not observe any serious side effects in the described patients. There were noticeable fluctuations of glucose and electrolyte levels surrounding pulse therapy, but they were not critical and could be effectively managed in a hospital setting. Heart rhythm disturbances, uncontrolled hypertension, and gastrointestinal bleeding were not registered in connection with pulse therapy. The period of viral shedding amounted to 20-23 days in the described patients. This correlates well with the data from other authors who found the average time to viral clearance in SARS-CoV-2 infection to be 20 (range: 17-24) days [2]. Hence, there was no evidence of protracted persistence of the virus in patients who received pulse therapy with high doses of methylprednisolone and IV immunoglobulin.

We used pulse therapy with methylprednisolone in combination with IV immunoglobulin in order to prevent infectious complications and based on immunoglobulin's own immunomodulating effect [12,16,18]. The effectiveness and safety of these components, either used separately or in combination, require further evaluation in randomized controlled studies.

## Conclusions

Pulse therapy with high doses of methylprednisolone and IV immunoglobulin was associated with a prompt elimination of respiratory failure, improvement in the clinical manifestations of the CRS, and reversal of pulmonary CT changes in patients with severe COVID-19 regardless of the initial level of biomarkers of inflammation. Pulse therapy with methylprednisolone in combination with IV immunoglobulin was generally safe and well-tolerated. There was no evidence of protracted persistence of the virus in patients who received this treatment.
